# Evaluation of Some Dental Myths Among the Adult Population in Riyadh, Saudi Arabia: A Cross-Sectional Study

**DOI:** 10.7759/cureus.33759

**Published:** 2023-01-13

**Authors:** Abdul Salam T.A., Vineet I Khinda, Ahmed M Alghamdi, Yazeed Z Alharthi, Hassan M Hodan, Muath H Binsuwaidan, Abdulaziz Z Alshathri, Muhannad Q Alanazi

**Affiliations:** 1 Dental Public Health, Department of Preventive Dental Science, College of Dentistry, King Saud Bin Abdulaziz University for Health Sciences, Riyadh, SAU; 2 Pediatric Dentistry, Department of Preventive Dental Science, College of Dentistry, King Saud Bin Abdulaziz University for Health Sciences, Riyadh, SAU; 3 General Dentistry, Department of Preventive Dental Science, College of Dentistry, King Saud Bin Abdulaziz University for Health Sciences, Riyadh, SAU

**Keywords:** oral health, dental education, dental misconceptions, dental misbeliefs, dental myths

## Abstract

Background

To provide effective oral health care to patients and healthy individuals, it is critical to recognize prevalent myths. Most myths cause patients to follow the wrong protocol in dentistry, which can make treatment difficult for the dentist. This study aimed to assess dental myths among the Saudi Arabian population in Riyadh.

Methodology

A descriptive cross-sectional questionnaire survey was conducted among Riyadh adults between August and October 2021. Saudi nationals aged 18-65 living in Riyadh without cognitive, hearing, or vision impairments and with limited or no trouble interpreting the questionnaire were surveyed. Only participants who consented to participate in the study were included. JMP Pro 15.2.0 was used to evaluate survey data. Frequency and percentage distributions were used for dependent and independent variables. The chi-square test evaluated the statistical significance of the variables, with a p-value of 0.05 being considered statistically significant.

Results

A total of 433 participants completed the survey. Half of the sample (50%) were aged 18-28; 50% were men; and 75% had a college degree. Higher-educated men and women did better on the survey. In particular, 80% of the participants believed “teething causes fever.” “Placing a (pain killer) tablet on a tooth reduces pain” was believed by 34.40% of the participants, and 26% thought pregnant women should not get dental work. Lastly, 79% of the participants believed that “infants obtain calcium from their mother’s teeth and bone.” Most sources of these pieces of information were online (62.60%).

Conclusions

Nearly half of the participants believe in dental health myths, and as a result, people follow unhealthy practices. This results in long-term health consequences. The government and health professionals must prevent the spread of such misconceptions. In this regard, dental health education may be helpful. Most of this study’s crucial findings are consistent with those of prior studies, indicating its accuracy.

## Introduction

Myths are beliefs, traditions, and cultural identities without a determinable basis or scientific explanation [1-3]. These are misconceptions that have become an integral part of the lives of people worldwide. A key setback of dental treatments is the myths that deter oral health-seeking activities [4]. Some myths highlight cultural differences and are hilariously obscure. One deception unearthed from the literature is that children with natal teeth develop wicked spiritual abilities [3].

Some common myths are that professional scaling leads to tooth mobility, sensitivity, and interdental spacing, the usage of stiff bristles, coal, or salt makes teeth whiter, one should not brush if the gingiva bleeds, the extracted teeth need not be replaced by artificial teeth as the latter are made of the teeth of other individuals [1,3,5]. A series of folk tales surrounding exfoliated teeth indicate the existence of various myths across the globe. Further, studies have revealed several myths such as those related to upper teeth extraction and its impact on vision; tobacco or alcohol consumption or application of clove oil alleviates toothache; and clefts are a result of prior sinful acts or exposure of expectant mothers to an eclipse [3,6].

According to a misconception, teething is attributed to fever, diarrhea, and ear infection [3,6,7]. The belief that tooth swelling should be fomented with warm water heedlessly leads to cellulitis [3]. Mothers should be aware that the eruption period of primary teeth correlates with the reduction of maternal immunity, enabling them to deal with potential childhood illnesses competently [7-9]. The misbelief of tooth extraction for a carious tooth indicated the unawareness of dental procedures among the general population. Albeit advances in modern dentistry, such false notions may substantially impact dental and general health [10].

Although numerous studies have been conducted in diverse populations to assess the prevalence of myths concerning oral health, only two have been conducted in Saudi Arabia. One of these two studies dealt only with the teething myths prevalent among mothers in Jazan Province [7,9]. Although Saudi Arabia has a high human development index, these myths are deep-rooted in the population, and the dearth of such studies in this region necessitates comprehensive research to be conducted in the capital city to alleviate the existing ignorance and cultural barriers. Hence, a need to assess the common myths regarding oral health becomes inevitable in order to promote awareness about the adverse effects of these practices. Thus, the study aimed to assess dental myths among the Saudi Arabian population in Riyadh.

## Materials and methods

Study design

A descriptive cross-sectional questionnaire survey was conducted among the adult population living in Riyadh, Kingdom of Saudi Arabia, from August to October 2021. The participants are Saudi nationals aged 18-65 years living in Riyadh, without a cognitive, hearing, or vision impairment, and with limited or no difficulty with interpreting the questionnaire. A total of 433 participants responded to the survey questionnaire. The participants who did not consent to participate in the study were excluded.

Research instrument

The questionnaire consisted of two sections: the first section included independent variables and demographic data such as age, gender, level of education, and source of information, and the second section included the dependent variables of the dental myth assessment. The second section consisted of 14 items, for which the participants indicated their agreement or disagreement [5]. A standardized questionnaire in the English language having good validity and reliability-a content validity ratio of 0.87 and internal consistency of 0.82, respectively-was used [5]. Excellent linguistic validity was obtained to administer the questionnaire to study participants in Arabic using the forward-backward-forward translation technique.

Ethics and informed consent

This study was approved by the Institutional Review Board (ethical number: IRBC/1471/21). The participants were clearly informed about the study’s objectives and provided formal informed consent. Additionally, participants were assured of the confidentiality of the information they provided in the survey questionnaire.

Data collection

Data were collected using a standardized web-based electronic survey using Google Forms. The Google Forms were distributed among the respondents through social media platforms such as Twitter, WhatsApp, and Telegram.

Statistical analysis

Data were entered into and analyzed using the JMP Pro 15.2.0 software. Descriptive and inferential statistics were used to represent the data. Descriptive statistics such as frequency and percentage distribution are used for dependent and independent variables. Inferential statistics such as the Chi-square test results indicated the distribution of dependent variables over independent variables. A p-value of <0.05 was considered statistically significant.

## Results

For ease of representation, descriptive statistics were to represent the demographic data in Table 1.

**Table 1:** Demographic characteristics of the participants.

Variable	Category	N	%
Age	18-28 years	217	50.10%
	29-38 years	92	21.20%
	39-48 years	67	15.50%
	49-58 years	45	10.40%
	59-65 years	12	2.80%
Gender	Female	207	47.80%
	Male	226	52.20%
Educational level	Primary school	2	0.50%
	Middle school	8	1.80%
	High school	98	22.60%
	University	325	75.10%
Source of information	Friends	180	41.60%
	Relatives	203	46.90%
	Television	107	24.70%
	Newspaper	35	8.10%
	Radio	30	6.90%
	Book	93	21.50%
	Internet (social media)	271	62.60%
	From a doctor	223	51.50%
	Others (personal experience)	36	8.30%
Participation	I agree to participate	433	100.00%

Considering a wide range of datasets for the research variables helps eliminate information gaps and, thus, enhance research reliability and validity to a great extent. Concerning age (Table 2), the chi-squared test revealed that around 54% of people aged 18-28 years disagree that using miswak only is enough, and there is no need for a toothbrush and paste (P = 0.001).

**Table 2:** Association of age groups with items in the questionnaire regarding dental myths. *: statistically significant

		Age										χ^2^ value	P-Value
		18-28 years		29-38 years		39-48 years		49-58 years		59-65 years			
		N	%	N	%	N	%	N	%	N	%		
1. Brushing with stiff bristles makes your teeth whiter.	Agree	31	45.60%	16	23.50%	13	19.10%	5	7.40%	3	4.40%	2.72	0.606
	Disagree	186	51.00%	76	20.80%	54	14.80%	40	11.00%	9	2.50%		
2. Using miswak only is enough, and there is no need for a toothbrush and paste.	Agree	26	32.90%	15	19.00%	21	26.60%	13	16.50%	4	5.10%	18.895	0.001*
	Disagree	191	54.00%	77	21.80%	46	13.00%	32	9.00%	8	2.30%		
3. When gums bleed, it is better not to brush your teeth.	Agree	69	59.50%	15	12.90%	11	9.50%	16	13.80%	5	4.30%	14.724	0.005*
	Disagree	148	46.70%	77	24.30%	56	17.70%	29	9.10%	7	2.20%		
4. Removal of calculus leads to the loosening of teeth.	Agree	44	51.80%	18	21.20%	16	18.80%	3	3.50%	4	4.70%	7.046	0.133
	Disagree	173	49.70%	74	21.30%	51	14.70%	42	12.10%	8	2.30%		
5. Using home stuff such as coal and salt makes your teeth whiter.	Agree	47	54.00%	17	19.50%	10	11.50%	12	13.80%	1	1.10%	3.84	0.428
	Disagree	170	49.10%	75	21.70%	57	16.50%	33	9.50%	11	3.20%		
6. Candy is the only cause of tooth decay.	Agree	24	43.60%	11	20.00%	12	21.80%	5	9.10%	3	5.50%	3.952	0.412
	Disagree	193	51.10%	81	21.40%	55	14.60%	40	10.60%	9	2.40%		
7. Decay is hereditary.	Agree	28	35.90%	28	35.90%	14	17.90%	6	7.70%	2	2.60%	14.507	0.006*
	Disagree	189	53.20%	64	18.00%	53	14.90%	39	11.00%	10	2.80%		
8. For toothache, the best treatment is tooth extraction.	Agree	25	55.60%	4	8.90%	10	22.20%	5	11.10%	1	2.20%	5.464	0.243
	Disagree	192	49.50%	88	22.70%	57	14.70%	40	10.30%	11	2.80%		
9. If you have pain in a particular tooth, placing a painkiller tablet on it reduces the pain.	Agree	89	59.70%	24	16.10%	20	13.40%	10	6.70%	6	4.00%	11.888	0.018*
	Disagree	128	45.10%	68	23.90%	47	16.50%	35	12.30%	6	2.10%		
10. There is no need to take care of baby teeth because they will fall off anyway.	Agree	14	73.70%	3	15.80%	2	10.50%	0	0.00%	0	0.00%	5.411	0.24
	Disagree	203	49.00%	89	21.50%	65	15.70%	45	10.90%	12	2.90%		
11. Placing of milk bottle inside the mouth of the baby during sleep does not harm teeth.	Agree	45	56.20%	13	16.20%	13	16.20%	6	7.50%	3	3.80%	3.058	0.548
	Disagree	172	48.70%	79	22.40%	54	15.30%	39	11.00%	9	2.50%		
12. Teething causes fever.	Agree	162	46.70%	80	23.10%	55	15.90%	39	11.20%	11	3.20%	9.154	0.057
	Disagree	55	64.00%	12	14.00%	12	14.00%	6	7.00%	1	1.20%		
13. No dental treatment should be done during pregnancy.	Agree	60	51.70%	31	26.70%	16	13.80%	8	6.90%	1	0.90%	6.556	0.161
	Disagree	157	49.50%	61	19.20%	51	16.10%	37	11.70%	11	3.50%		
14. During pregnancy, the baby absorbs calcium from the teeth and bones of their mother.	Agree	161	47.10%	76	22.20%	58	17.00%	36	10.50%	11	3.20%	7.24	0.124
	Disagree	56	61.50%	16	17.60%	9	9.90%	9	9.90%	1	1.10%		

Similarly, the majority of participants in the 18-28 age group disagreed that it is better not to brush the teeth when gums bleed (P = 0.005); placing a painkiller tablet on a tooth that is aching will relieve pain (P = 0.018), and decay is hereditary (P = 0.006).

Concerning gender (Table 3), the chi-squared test revealed that the majority of male participants agreed that brushing with stiff bristles makes one’s teeth whiter (64.7%; P = 0.024); it is better to not brush your teeth when gums bleed (70.7%; P = 0.001); placing a tablet on the paining tooth will relieve the pain (61.1%; P = 0.007); there is no need to take care of baby teeth because they will fall off anyway (84.2%; P = 0.004); placing of milk bottle inside the mouth of the baby during sleep does not harm the teeth (62.5%; P = 0.041); and no dental treatment should be done during pregnancy (62.1%; P = 0.013). In contrast, the majority of female participants agreed that using miswak only is enough, and there is no need for a toothbrush and paste (59.5%; P = 0.021); decay is hereditary (66.6%; P = 0.001); teething causes fever (52.4%; P = 0.001); and during pregnancy, the baby absorbs calcium from the teeth and bones of their mother (52.3%; P = 0.001).

**Table 3:** Association of gender with items in the questionnaire regarding dental myths. *: statistically significant

		Gender				χ^2^ value	P-Value
		Female		Male			
		N	%	N	%		
1. Brushing with stiff bristles makes your teeth whiter.	Agree	24	35.30%	44	64.70%	5.061	0.024*
	Disagree	183	50.10%	182	49.90%		
2. Using miswak only is enough, and there is no need for a toothbrush and paste.	Agree	47	59.50%	32	40.50%	5.29	0.021*
	Disagree	160	45.20%	194	54.80%		
3. When gums bleed, it is better not to brush your teeth.	Agree	34	29.30%	82	70.70%	21.723	0.001*
	Disagree	173	54.60%	144	45.40%		
4. Removal of calculus leads to the loosening of teeth.	Agree	37	43.50%	48	56.50%	0.775	0.379
	Disagree	170	48.90%	178	51.10%		
5. Using home stuff such as coal and salt makes your teeth whiter.	Agree	40	46.00%	47	54.00%	0.146	0.702
	Disagree	167	48.30%	179	51.70%		
6. Candy is the only cause of tooth decay.	Agree	28	50.90%	27	49.10%	0.243	0.622
	Disagree	179	47.40%	199	52.60%		
7. Decay is hereditary.	Agree	52	66.70%	26	33.30%	13.563	0.001*
	Disagree	155	43.70%	200	56.30%		
8. For toothache, the best treatment is tooth extraction.	Agree	18	40.00%	27	60.00%	1.226	0.268
	Disagree	189	48.70%	199	51.30%		
9. If you have pain in a particular tooth, placing a painkiller tablet on it reduces the pain.	Agree	58	38.90%	91	61.10%	7.179	0.007*
	Disagree	149	52.50%	135	47.50%		
10. There is no need to take care of baby teeth because they will fall off anyway.	Agree	3	15.80%	16	84.20%	8.164	0.004*
	Disagree	204	49.30%	210	50.70%		
11. Placing of milk bottle inside the mouth of the baby during sleep does not harm teeth.	Agree	30	37.50%	50	62.50%	4.177	0.041*
	Disagree	177	50.10%	176	49.90%		
12. Teething causes fever.	Agree	182	52.40%	165	47.60%	15.098	0.001*
	Disagree	25	29.10%	61	70.90%		
13. No dental treatment should be done during pregnancy.	Agree	44	37.90%	72	62.10%	6.192	0.013*
	Disagree	163	51.40%	154	48.60%		
14. During pregnancy, the baby absorbs calcium from the teeth and bones of their mother.	Agree	179	52.30%	163	47.70%	13.402	0.001*
	Disagree	28	30.80%	63	69.20%		

Regarding educational level (Table 4), the chi-squared test revealed that most university-educated participants disagreed that using miswak only is enough, and there is no need for a toothbrush and paste (77.7%; P = 0.035).

**Table 4:** Association of educational level with items in the questionnaire regarding dental myths. *: statistically significant

		Educational level								Chi-squared value	P-value
		High school		Middle school		Primary school		University			
		N	%	N	%	N	%	N	%		
1. Brushing with stiff bristles makes your teeth whiter.	Agree	14	20.60%	0	0.00%	0	0.00%	54	79.40%	2.216	0.529
	Disagree	84	23.00%	8	2.20%	2	0.50%	271	74.20%		
2. Using miswak only is enough, and there is no need for a toothbrush and paste.	Agree	25	31.60%	3	3.80%	1	1.30%	50	63.30%	8.591	0.035*
	Disagree	73	20.60%	5	1.40%	1	0.30%	275	77.70%		
3. When gums bleed, it is better not to brush your teeth.	Agree	33	28.40%	2	1.70%	1	0.90%	80	69.00%	3.714	0.294
	Disagree	65	20.50%	6	1.90%	1	0.30%	245	77.30%		
4. Removal of calculus leads to the loosening of teeth.	Agree	29	34.10%	3	3.50%	0	0.00%	53	62.40%	10.546	0.014*
	Disagree	69	19.80%	5	1.40%	2	0.60%	272	78.20%		
5. Using home stuff such as coal and salt makes your teeth whiter.	Agree	17	19.50%	1	1.10%	1	1.10%	68	78.20%	2.001	0.572
	Disagree	81	23.40%	7	2.00%	1	0.30%	257	74.30%		
6. Candy is the only cause of tooth decay.	Agree	23	41.80%	2	3.60%	0	0.00%	30	54.50%	15.16	0.002*
	Disagree	75	19.80%	6	1.60%	2	0.50%	295	78.00%		
7. Decay is hereditary.	Agree	17	21.80%	1	1.30%	1	1.30%	59	75.60%	1.584	0.663
	Disagree	81	22.80%	7	2.00%	1	0.30%	266	74.90%		
8. For toothache, the best treatment is tooth extraction.	Agree	15	33.30%	2	4.40%	0	0.00%	28	62.20%	5.708	0.127
	Disagree	83	21.40%	6	1.50%	2	0.50%	297	76.50%		
9. If you have pain in a particular tooth, placing a painkiller tablet on it reduces the pain.	Agree	41	27.50%	0	0.00%	1	0.70%	107	71.80%	7.126	0.068
	Disagree	57	20.10%	8	2.80%	1	0.40%	218	76.80%		
10. There is no need to take care of baby teeth because they will fall off anyway.	Agree	5	26.30%	0	0.00%	0	0.00%	14	73.70%	0.583	0.9
	Disagree	93	22.50%	8	1.90%	2	0.50%	311	75.10%		
11. Placing of milk bottle inside the mouth of the baby during sleep does not harm teeth.	Agree	17	21.20%	1	1.20%	1	1.20%	61	76.20%	1.611	0.657
	Disagree	81	22.90%	7	2.00%	1	0.30%	264	74.80%		
12. Teething causes fever.	Agree	84	24.20%	6	1.70%	0	0.00%	257	74.10%	10.347	0.016*
	Disagree	14	16.30%	2	2.30%	2	2.30%	68	79.10%		
13. No dental treatment should be done during pregnancy.	Agree	34	29.30%	2	1.70%	2	1.70%	78	67.20%	9.89	0.02*
	Disagree	64	20.20%	6	1.90%	0	0.00%	247	77.90%		
14. During pregnancy, the baby absorbs calcium from the teeth and bones of their mother.	Agree	81	23.70%	7	2.00%	1	0.30%	253	74.00%	2.41	0.492
	Disagree	17	18.70%	1	1.10%	1	1.10%	72	79.10%		

Participants with university education also disagreed with the following: removal of calculus leads to the loosening of teeth (78.2%; P = 0.014); Candy is the only cause of tooth decay (78%; P = 0.002); teething causes fever (79.1%; P = 0.016); and no dental treatment should be done during pregnancy (77.9%; P = 0.02).

Regarding the source of information (Table 5), the chi-squared test revealed that when the source of information is the Internet (social media), participants disagreed that it is better to not brush the teeth when gums bleed (63.3%; P = 0.001); using home stuff such as coal and salt makes teeth whiter (63.3%; P = 0.008); candy is the only cause of tooth decay (64.8%; P = 0.001); for toothache, the best treatment is extraction (63.4%; P = 0.014); if a particular tooth is paining, placing a painkiller tablet on that tooth reduces the pain (64.1%; P = 0.004); there is no need to take care of baby teeth because they will fall off anyway (63.8%; P = 0.001); and teething causes fever (59.3%) (P = 0.013).

**Table 5:** Association of the source of information with items in the questionnaire regarding dental myths. *: statistically significant

			Source of information									χ^2^ value	P-Value
			Relatives	Friends	TV	Newspaper	Radio	Book	Internet (social media)	From a doctor	Others (personal experience)		
1. Brushing with stiff bristles makes your teeth whiter.	Agree	N	35	32	12	7	6	11	42	27	4	11.313	0.255
		%	51.50%	47.10%	17.60%	10.30%	8.80%	16.20%	61.80%	39.70%	5.90%		
	Disagree	N	168	148	95	28	24	82	229	196	32		
		%	46.00%	40.50%	26.00%	7.70%	6.60%	22.50%	62.70%	53.70%	8.80%		
2. Using miswak only is enough, and there is no need for a toothbrush and paste.	Agree	N	41	35	22	6	4	16	44	45	5	6.035	0.736
		%	51.90%	44.30%	27.80%	7.60%	5.10%	20.30%	55.70%	57.00%	6.30%		
	Disagree	N	162	145	85	29	26	77	227	178	31		
		%	45.80%	41.00%	24.00%	8.20%	7.30%	21.80%	64.10%	50.30%	8.80%		
3. When gums bleed, it is better not to brush your teeth.	Agree	N	49	54	17	8	9	19	64	44	11	30.199	0.001*
		%	42.20%	46.60%	14.70%	6.90%	7.80%	16.40%	55.20%	37.90%	9.50%		
	Disagree	N	154	126	90	27	21	74	207	179	25		
		%	48.60%	39.70%	28.40%	8.50%	6.60%	23.30%	65.30%	56.50%	7.90%		
4. Removal of calculus leads to the loosening of teeth.	Agree	N	41	37	19	6	9	14	51	34	7	10.384	0.32
		%	48.20%	43.50%	22.40%	7.10%	10.60%	16.50%	60.00%	40.00%	8.20%		
	Disagree	N	162	143	88	29	21	79	220	189	29		
		%	46.60%	41.10%	25.30%	8.30%	6.00%	22.70%	63.20%	54.30%	8.30%		
5. Using home stuff such as coal and salt makes your teeth whiter.	Agree	N	50	43	17	5	4	12	52	34	6	22.143	0.008*
		%	57.50%	49.40%	19.50%	5.70%	4.60%	13.80%	59.80%	39.10%	6.90%		
	Disagree	N	153	137	90	30	26	81	219	189	30		
		%	44.20%	39.60%	26.00%	8.70%	7.50%	23.40%	63.30%	54.60%	8.70%		
6. Candy is the only cause of tooth decay.	Agree	N	24	28	8	2	2	5	26	18	0	35.409	0.001*
		%	43.60%	50.90%	14.50%	3.60%	3.60%	9.10%	47.30%	32.70%	0.00%		
	Disagree	N	179	152	99	33	28	88	245	205	36		
		%	47.40%	40.20%	26.20%	8.70%	7.40%	23.30%	64.80%	54.20%	9.50%		
7. Decay is hereditary.	Agree	N	34	30	14	5	4	18	47	42	8	5.013	0.833
		%	43.60%	38.50%	17.90%	6.40%	5.10%	23.10%	60.30%	53.80%	10.30%		
	Disagree	N	169	150	93	30	26	75	224	181	28		
		%	47.60%	42.30%	26.20%	8.50%	7.30%	21.10%	63.10%	51.00%	7.90%		
8. For a toothache, the best treatment is tooth extraction.	Agree	N	22	22	5	3	2	6	25	14	1	20.637	0.014*
		%	48.90%	48.90%	11.10%	6.70%	4.40%	13.30%	55.60%	31.10%	2.20%		
	Disagree	N	181	158	102	32	28	87	246	209	35		
		%	46.60%	40.70%	26.30%	8.20%	7.20%	22.40%	63.40%	53.90%	9.00%		
9. If you have pain in a particular tooth, placing a painkiller tablet on that tooth reduces the pain.	Agree	N	75	73	33	11	9	28	89	63	5	24.292	0.004*
		%	50.30%	49.00%	22.10%	7.40%	6.00%	18.80%	59.70%	42.30%	3.40%		
	Disagree	N	128	107	74	24	21	65	182	160	31		
		%	45.10%	37.70%	26.10%	8.50%	7.40%	22.90%	64.10%	56.30%	10.90%		
10. There is no need to take care of baby teeth because they will fall off anyway.	Agree	N	12	12	1	1	2	2	7	2	1	31.216	0.001*
		%	63.20%	63.20%	5.30%	5.30%	10.50%	10.50%	36.80%	10.50%	5.30%		
	Disagree	N	191	168	106	34	28	91	264	221	35		
		%	46.10%	40.60%	25.60%	8.20%	6.80%	22.00%	63.80%	53.40%	8.50%		
11. Placing of milk bottle inside the mouth of the baby during sleep does not harm teeth.	Agree	N	45	34	14	6	5	11	52	32	5	15.815	0.071
		%	56.20%	42.50%	17.50%	7.50%	6.20%	13.80%	65.00%	40.00%	6.20%		
	Disagree	N	158	146	93	29	25	82	219	191	31		
		%	44.80%	41.40%	26.30%	8.20%	7.10%	23.20%	62.00%	54.10%	8.80%		
12. Teething causes fever.	Agree	N	172	154	90	31	28	79	220	183	30	20.912	0.013*
		%	49.60%	44.40%	25.90%	8.90%	8.10%	22.80%	63.40%	52.70%	8.60%		
	Disagree	N	31	26	17	4	2	14	51	40	6		
		%	36.00%	30.20%	19.80%	4.70%	2.30%	16.30%	59.30%	46.50%	7.00%		
13. No dental treatment should be done during pregnancy.	Agree	N	57	52	26	11	10	20	70	54	9	6.231	0.717
		%	49.10%	44.80%	22.40%	9.50%	8.60%	17.20%	60.30%	46.60%	7.80%		
	Disagree	N	146	128	81	24	20	73	201	169	27		
		%	46.10%	40.40%	25.60%	7.60%	6.30%	23.00%	63.40%	53.30%	8.50%		
14. During pregnancy, the baby absorbs calcium from the teeth and bones of his mom	Agree	N	168	147	90	30	26	71	217	175	32	12.461	0.189
		%	49.10%	43.00%	26.30%	8.80%	7.60%	20.80%	63.50%	51.20%	9.40%		
	Disagree	N	35	33	17	5	4	22	54	48	4		
		%	38.50%	36.30%	18.70%	5.50%	4.40%	24.20%	59.30%	52.70%	4.40%		

## Discussion

This study aimed to assess the prevalence of myths among the Saudi Arabian population in Riyadh; the research findings have provided strategic information about the myths. As a critical finding, it has been revealed that people aged 18 to 65 years have different myths with no evidence in dentistry. According to Athavale et al. [11], the notion that candy consumption is the only reason for tooth decay is completely wrong. Myths can spread throughout a population for various reasons, including inadequate education, cultural beliefs, and societal misconceptions. Freeman et al. [12] claimed that people believe in most myths due to their lack of dental health knowledge, which often leads to engagement in dental activities that can be detrimental to the gum or the teeth [13-19]. As per Smith et al. [17], key research findings can be analyzed through a proper investigation and assessment with a consideration of the discussion areas.

In this study, 15.7% of the participants believed that brushing with stiff bristles makes the teeth whiter. The participants were unaware that it is important to use a proper brushing technique, rather than forceful brushing, which leads to abrasion of the teeth. The prevalence of this myth was less prevalent in this study compared to a similar one done in a different region in Saudi Arabia, Taif City, by Al-Harthi et al. [5], who found a prevalence rate of 31% in their sample. Moreover, Vignesh and Priyadarshni [1] studied the prevalence of dental myths in Maduravoyal and found that 70% of the sample believed in this particular myth [1]. Around 18% of the sample in this study believed that using miswak only is enough, and there is no need for a toothbrush and paste, whereas the remaining 82% preferred to use toothpaste. This finding was significantly related to the gender of the participants. The prevalence of this myth in this study was less than that found in the study in Taif City, where 34% of the sample preferred using Miswak and 66% preferred using toothpaste. Moreover, compared to a study done in India, where 57% preferred using tree sticks, 43% opted for using toothpaste for cleaning the teeth [1,5]. Miswak has potent antimicrobial and antiplaque substances; however, its use can cause trauma to the gingiva [1]. In this study, 26.8% of the respondents believed that when gums bleed, it is better not to brush their teeth. This belief is common because of the notion that brushing provokes bleeding; however, most were unaware that this is not the real reason. The prevalence of this myth is lower in this study compared to a study conducted in Taif City, where more than half of the respondents (56%) believed in this myth, as well as another study conducted in Maduravoyal, where this percentage was 51.6% [1,5]. The main reason behind these differences is the educational background of the samples used. In this study, most participants had a university-level educational background. Their high educational level helped them understand that using only miswak is not enough.

In this study, 80.40% of the participants and, in the study in Taif City, 66.9% of the sample disagreed that the removal of calculus leads to the loosening of the teeth. This consistent finding indicated that people have become aware of dental procedures due to various school awareness camps. This finding, however, was consistent with previous studies by Ain et al. [20], where 72.7% believed in this myth, and Mary et al. [15], where 72.8% believed in this myth. A minor percentage of participants (20.10%) in this study believed that using home stuff such as coal and salt makes the teeth whiter. This finding is consistent with the study by Saravanan and Thirineevannan [21], where only 10% believed in this myth, as well as the study by Al-Harthi et al. [5], where 44% believed in this myth. Household substances such as salt and coal can clean teeth. However, their teeth-whitening effects have not been proven yet. In addition, excess use of salt and coal can negatively affect the health of the user, causing damage (abrasion) to the inorganic and organic parts of the teeth as well as high blood pressure. According to Rasul et al. [16], such practices must be avoided. Further, 87.3% of the participants in this study and 75% of those in the study by Al-Harthi et al. [5] did not believe that candy is the only cause of tooth decay. This consistent finding indicated that health professionals are not restricted to advising against the consumption of candy; they have started educating their patients that dental caries is a common disease that can be considered “complex” or multi-factorial.

It was found that 82% and 75% of the participants in this study and Al-Harthi et al. [5], respectively, did not believe that decay is hereditary. Dental myths, including tooth decays are caused by worms and brushing and scaling cause the teeth to become more mobile, have evolved over ages and can lead to a greater refrain from seeking dental treatments as well as follow-up by patients. This indicates that the participants were aware of oral health. This myth was less prevalent in this study compared to that by Gambhir [22], where 62.7% believed in it. About 89.60% and 88% of the participants in this study and the study by Al-Harthi et al. [5], respectively, did not believe that the best treatment for toothache is tooth extraction. This indicates that the participants were aware of different dental procedures to save a tooth, such as the use of fillings and root canal treatment. This myth was less prevalent in this study compared to Ain et al. [20], where 59.6% believed this myth. According to Parahoo et al. [14], the belief that the best treatment for tooth pain is extraction is completely wrong. Around 34.40% and 49% of the participants in this study and in Al-Harthi et al. [5], respectively, believed that “if you have pain in a particular tooth, placing a pain killer tablet on that tooth can reduce the pain.” In Ain et al. [20], 75% of the participants believed that placing clove over a particular tooth could reduce pain. Regarding the importance of deciduous teeth, only 4.40% in this present study and 12% in Al-Harthi et al. [5] believed that there is no need to take care of baby teeth because they will fall off anyway. Clearly, the participants were aware of the importance of baby teeth in chewing, aesthetics, and the maintenance of the space for the permanent teeth that will erupt. This myth was less prevalent in this study compared to Gambhir et al. [22], where 52% of the participants believed this myth. Around 18% and 35% of the participants in this study and Al-Harthi et al. [5], respectively, believed that placing of milk bottle inside the mouth of the baby during sleep does not harm the teeth. The participants were unaware that bacteria could thrive on the lactose, causing caries. In contrast, about 78% of the participants in Ain et al. [20] opined that a child’s milk teeth need not be cleaned [20]. More than half of the participants in this study (80%) and in Al-Harthi et al. [5] (69%) believed that teething causes, which was almost similar to the percentage reported by Ain et al. [20] (63%).

When asked about dental treatments during pregnancy, about 26% and 34% of the participants in this study and Al-Harthi et al. [5] believed that no dental treatment should be done during this period. This could be because undergoing dental treatment during pregnancy would affect the development of the fetus. However, they should be aware that treatment can be done in the case of an emergency and other types of procedures can be done after the second trimester [22]. The prevalence of this myth was less as compared to Gambhir et al. [22], where 71% believed in it. The findings of this study revealed that age, gender, and level of education impacted participants’ perceptions of myths concerning dentistry. It was noted that younger individuals had a more positive perception than older individuals. Surprisingly, more educated participants responded more positively toward the perception of dental-related myths compared to Al-Harthi et al. [5]. A survey report from Pakistan noted that more illiterates and older people believed in one or more dental myths [23]. This finding suggests that delivering a sound education system to all age groups will alleviate the population's ignorance about myths and help people overcome these cultural barriers. This study did not shed light on the practices adopted and avoided myths about better dental health. Hence, it can be considered one of this study's potential weaknesses. Moreover, most of the findings were negative because people in Riyadh believe in myths; thus, it is essential to conduct proper health campaigns to develop awareness about dental health issues among the population.

Limitations

This study was based on the responses of normal individuals; no detailed answers from dental experts were considered in this study. Hence, the selection of this inadequate sample could affect the study outcomes. Moreover, selection bias might also be an issue in this research, causing reliability and validity issues in the study. As stated by Löhr et al. [13], considering both qualitative and quantitative data together can provide an opportunity to cross-check or reverify the information. Since only quantitative data were not in this research, the data could not be qualitatively reverified, which also affected reliability. In the future, a qualitative study can be conducted, and the outcomes can be applied to the results of this study to ensure better research effectiveness. A pilot study was not conducted before the execution of the final research. Thus, the responsiveness to the methodological instrument, that is, the questionnaire, implemented in this research could not be tested or retested. This affected the study's reliability and validity since the data collection instrument was not checked according to the research criteria.

## Conclusions

This study confirmed that dental myths related to brushing habits, factors causing caries, and other aspects are quite prevalent among the population. Thus, people must refrain from spreading such myths and seek regular advice from a registered dentist. The government should also implement legislation to prevent such occurrences. It will restrict the formation of a wrong idea, which will benefit societal wellness. It is recommended that doctors, medical practitioners, health employees, and the government encourage individuals to take care of their dental health and learn more about it in order to eliminate myths from society to a great level.

## Appendices

The data was collected using a standardized web-based electronic survey available at https://figshare.com/articles/dataset/Dental_Myths_Responses_xlsx/21717308.
